# Supplementary material for: Vancomycin Drug Reaction with Eosinophilia and Systemic Symptoms: Meta-Analysis and Pharmacovigilance Study
Source: J Clin Med. 2025 Jan 31;14(3):930. doi: 10.3390/jcm14030930 (PMC11818417; doi:10.3390/jcm14030930)
Supplement: Supplementary file 1 [file jcm-14-00930-s001.zip › jcm-3450208-supplementary.pdf]

Supplementary:

Table S1: Search strategy PICO (Population Intervention Comparability Outcome)

Table S2: FAERS database Standardized MedDRA Queries (SMQs)

Figure S1: Subgroup if allele frequency>0.0036.

Figure S2: Risk Assessment of Studies Included in Meta-analysis JBI Checklist Synthesized by Revman 5.3

Figure S3: Doi Plot for Publication Bias Generated by STATA 18

Table S3: GRADE Assessment

Table S4: Summary of Demographic Characteristics FAERS Reports

Table S1: Search strategy PICO

Population	Intervention	Comparison	Outcome
Drug-induced Drug reaction with eosinophilia and systemic symptoms syndrome	Vancomycin	Sulfonamides	Drug reaction with eosinophilia and systemic symptoms Hospitalization
Drug-induced DRESS	Glycopeptide antimicrobial	Sulfamethoxazole	Serious cutaneous adverse reaction
Antibiotic-induced DRESS	Vancomycin antibiotic	Sulfamethoxazole trimethoprim	Drug reaction with eosinophilia and systemic symptoms
Antibiotic-induced Drug hypersensitivity syndrome	Vancomycin antimicrobial	Co-trimoxazole	Drug reaction with eosinophilia and systemic symptoms syndrome
Antimicrobial DRESS	Vancomycin hydrochloride	Penicillin	DRESS
Antibiotic-induced Drug hypersensitivity syndrome	Glycopeptide antibiotic	Quinolones	DRESS syndrome
Antibiotic-induced sCARS		Levofloxacin	Drug hypersensitivity syndrome
Antibiotic and cutaneous severe adverse reactions		Ciprofloxacin	Safety
Antimicrobial-induced sCARS		Cephalosporins	adverse effects
Cohort		Ceftriaxone	side effects
Observational study		Cefepime	adverse reaction
prospective study		Antiseizure medications	Hospitalization

Population-based cohort		Carbamazepine	ADR
retrospective study		Lamotrigine	adverse drug reaction
HLA-A*32:01 DRESS		Anti-epileptics	severe adverse reaction
HLA-A*32:01 Drug-induced Drug reaction with eosinophilia and systemic symptoms		Phenytoin	HLA induced adverse events
HLA-A*32:01 hypersensitivity reaction		Abacavir	HLA-A*32:01 induced adverse events
HLA Drugs		Dapsone	HLA-A*32:01 induced adverse events
Case-control study		Sulfasalazine	HLA-A*32:01 Drug-induced Drug reaction with eosinophilia and systemic symptoms
Case series		Sulphonamides	Liver injury and eosinophilia
Allele frequency DRESS		NSAIDs	
Allele Drug-induced Drug reaction with eosinophilia and systemic symptoms		Minocycline	
Registry		Tetracycline	
Communication		Doxycycline	
Letter to the editor		Non-steroidal anti-inflammatory drugs	

Table S2: FAERS database Standardized MedDRA Queries (SMQs)

Drug Name	MedDRA term
Vancomycin	Drug reaction with eosinophilia and systemic symptoms
Vancomycin hydrochloride	Drug induced hypersensitivity syndrome
Sulfamethoxazole	Drug rash with eosinophilia and systemic syndrome
Sulfamethoxazole and trimethoprim	Hypersensitivity syndrome
Sulfamethoxazole/trimethoprim	DRESS syndrome
Ciprofloxacin	
Ciprofloxacin hydrochloride	
Ciprofloxacin lactate	
Levofloxacin	
Levofloxacin anhydrous	
Levofloxacin hydrochloride	
Piperacillin	
Piperacillin/tazobactam	
Piperacillin sodium/tazobactam sodium	
Ceftriaxone	
Ceftriaxone sodium	
Cefepime hydrochloride	
Ceftazidime sodium	
Ceftazidime	

Figure S1: Risk Assessment of Studies Included in Meta-analysis JBI Checklist Synthesized by Revman

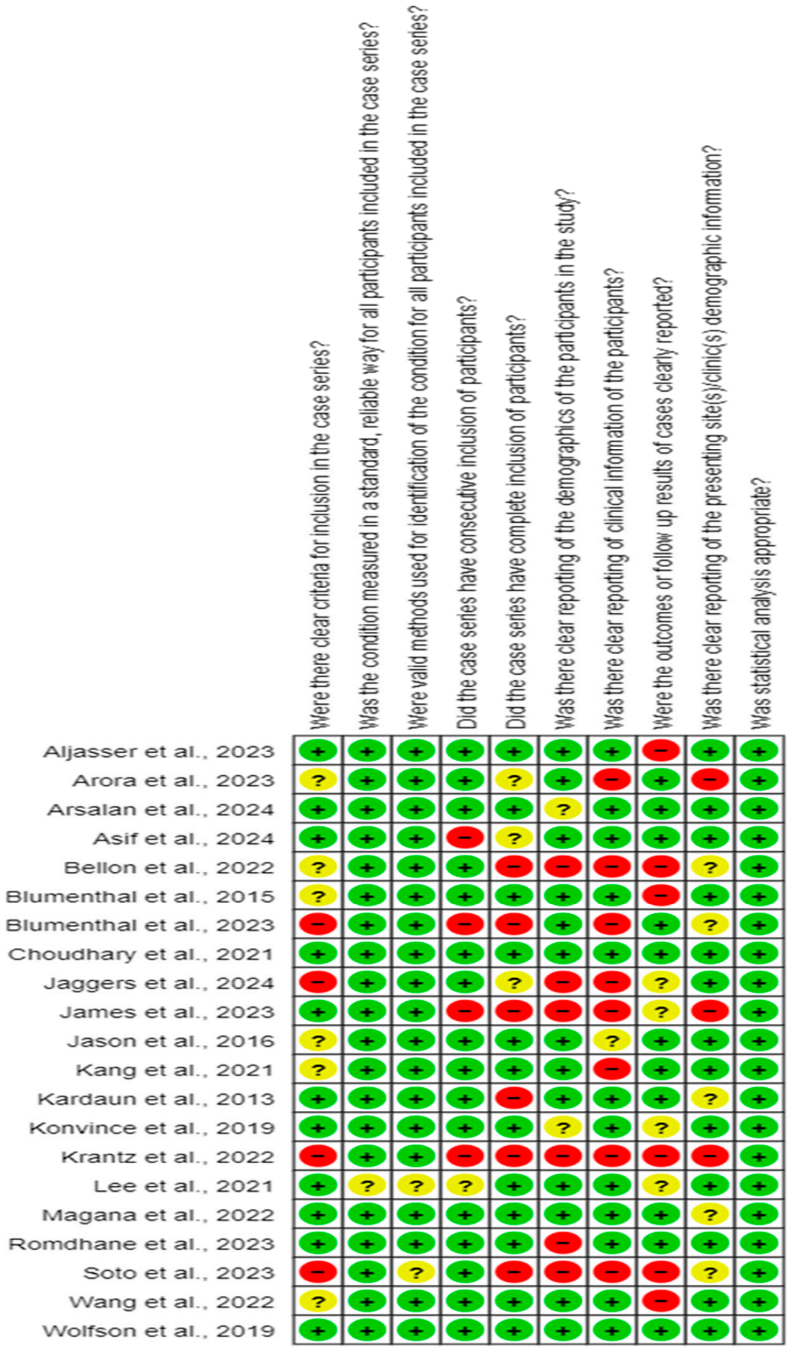

Figure S2: Subgroup if allele frequency>0.0036, 4=Caucasians

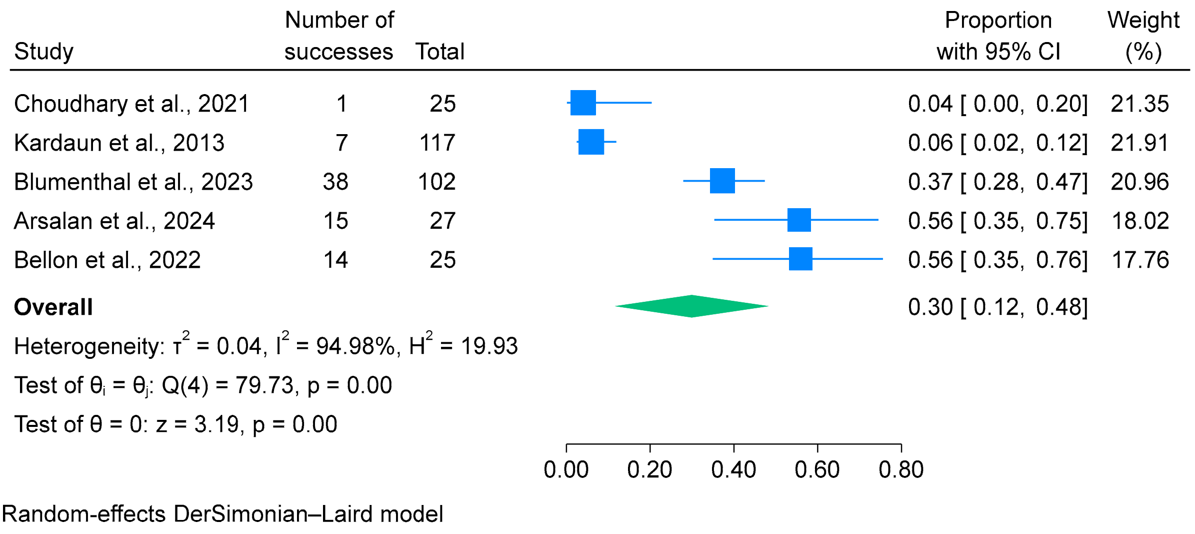

Figure S3: Doi Plot for Publication Bias Generated by STATA 18

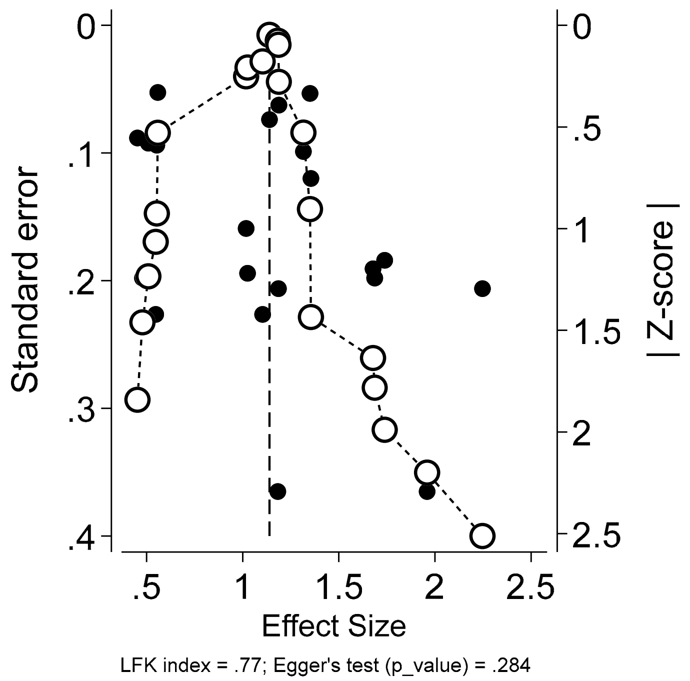

Table S3: GRADE assessment

No. of studies	Risk of Bias	Imprecision	Inconsistency	Indirectness	Publication bias	Certainty
21	low	No	high	No	Not detected	Moderate

Table S4: Summary of Demographic Characteristics FAERS Reports.

	Vancomycin<br>n=2558	Sulfonamides<br>n=2306	Cephalosporins<br>n=775	Quinolones<br>n=752	Piperacillin<br>n=1238	p-value
Mean age±SD	50.9±0.44	49.1±0.56	50±0.91*	56.3±0.80	54.6±0.10	<0.01
Females (%)	1006 (43.6%)	613 (41.1%)	312 (46.7)	317 (49.1%)	483 (39%)	0.38
Median Concomitant antimicrobials (range)	1 (0-6)	0 (0-6)	0 (0-5)	0 (0-4)	0 (0-5)	<0.01

*p-value=0.36
